# Chiral exceptional point enhanced active tuning and nonreciprocity in micro-resonators

**DOI:** 10.1038/s41377-024-01686-w

**Published:** 2025-01-09

**Authors:** Hwaseob Lee, Lorry Chang, Ali Kecebas, Dun Mao, Yahui Xiao, Tiantian Li, Andrea Alù, Sahin K. Özdemir, Tingyi Gu

**Affiliations:** 1 Department of Electrical and Computer Engineering, University of Delaware, Newark, Delaware 19716 USA; 2 Department of Engineering Science and Mechanics, Pennsylvania State University, University Park, PA 16802 USA; 3 Photonics Initiative, Advanced Science Research Center, City University of New York, New York, NY 10031 USA; 4 Physics Program, Graduate Center, City University of New York, New York, NY 10016 USA; 5 Department of Electrical and Computer Engineering, Saint Louis University, Saint Louis, MO 63103 USA

**Keywords:** Microresonators, Nonlinear optics

## Abstract

Exceptional points (EPs) have been extensively explored in mechanical, acoustic, plasmonic, and photonic systems. However, little is known about the role of EPs in tailoring the dynamic tunability of optical devices. A specific type of EPs known as chiral EPs has recently attracted much attention for controlling the flow of light and for building sensors with better responsivity. A recently demonstrated route to chiral EPs via lithographically defined symmetric Mie scatterers on the rim of resonators has not only provided the much-needed mechanical stability for studying chiral EPs, but also helped reduce losses originating from nanofabrication imperfections, facilitating the in-situ study of chiral EPs and their contribution to the dynamics and tunability of resonators. Here, we use asymmetric Mie scatterers to break the rotational symmetry of a microresonator, to demonstrate deterministic thermal tuning across a chiral EP, and to demonstrate EP-mediated chiral optical nonlinear response and efficient electro-optic tuning. Our results indicate asymmetric electro-optic modulation with up to 17 dB contrast at GHz and CMOS-compatible voltage levels. Such wafer-scale nano-manufacturing of chiral electro-optic modulators and the chiral EP-tailored tunning may facilitate new micro-resonator functionalities in quantum information processing, electromagnetic wave control, and optical interconnects.

## Abstract (web summary)

CMOS wafer scale manufactured chiral exceptional point non-Hermitian modulators were demonstrated through AIM photonics. This enables GHz-speed asymmetric electro-optic modulation with 17 dB contrast using CMOS-compatible voltages.

## Graphical abstract

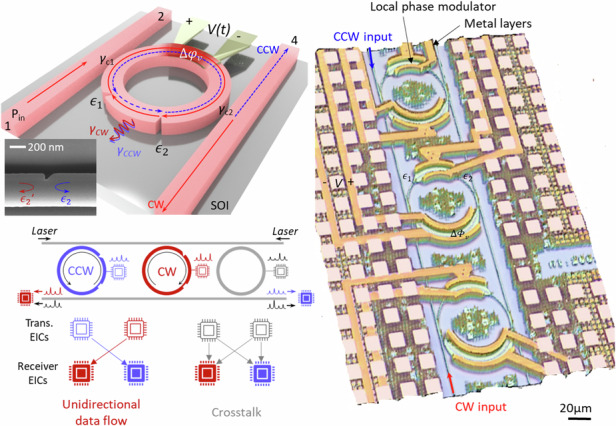

## Introduction

Non-Hermitian spectral singularities known as exceptional points (EPs), and the associated reduction in a system’s dimensionality, have been extensively studied in mechanical, acoustic, plasmonic, and nanophotonic systems for their exotic response^1–3^. A specific type of EPs known as chiral EPs has been observed in ring resonators by properly positioning scatterers to perturb traveling wave resonator modes^4–7^ or by terminating one end of a waveguide with a mirror^8,9^. Chirality here refers to the direction of rotation of the optical field inside the resonator. At a chiral EP the modal fields propagating in the clockwise (CW) or counterclockwise (CCW) direction become degenerate.^4^ EPs can also emerge in parity-time (PT) symmetric systems by judiciously engineering the imaginary part of the refractive index of subsystems and their coupling. This corresponds to tuning the gain-loss balance of subsystems in active PT systems^10–14^ and the loss-imbalance between subsystems in passive PT systems^15–18^. EPs have also been demonstrated in various quantum systems, including atomic ensembles^19^, single spins^20^, single trapped ions^21^, and superconducting qubits^14^. Recently, electrostatic tuning of graphene permittivity has been implemented to achieve topological control of terahertz light across EPs^22,23^. The utility of EPs for achieving highly tunable systems, optical modulators, and enhanced light-matter interactions has not been studied thoroughly and it remains elusive. Elucidating this can potentially contribute to energy-efficient photonic precision instrumentations, such as analog processors^24^, gyroscopes^25^, and atomic clocks.

**Fig. 1:**
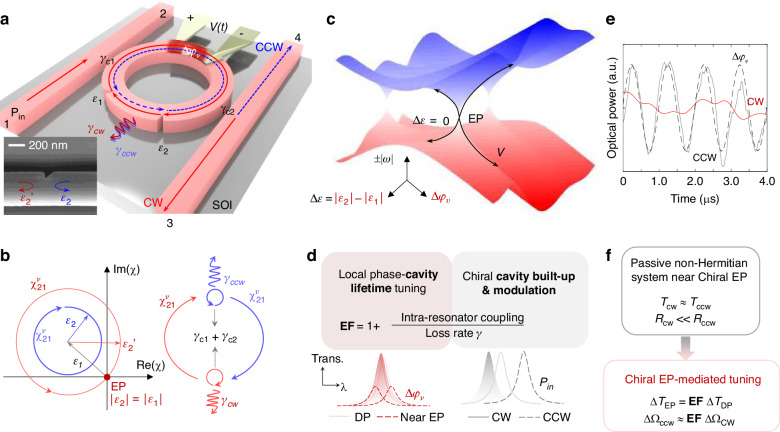
Broken rotational symmetry mediated active tuning. **a** Schematics of the chiral microring resonator (MRR), with lithographically defined low loss asymmetric Mie scatterer (Inset), which mediates asymmetric coupling between clockwise (CW) and counterclockwise (CCW) modes. **b** Asymmetric coupling strength ($|{\chi }_{12}^{v}|$ and $|{\chi }_{21}^{v}|$) tuned by the phase (∆*φ*_*v*_) determined by the optical path between Mie-scatterers. An exceptional point (EP) emerges when the perturbation strengths of Mie scatterers are matched ($\varDelta \epsilon$ = |${\epsilon }_{1}$ | - |${\epsilon }_{2}$ | = 0). The complex inter-mode coupling contributes to the photon loss rate of the CW or CCW modes and is tunable through the inter-scatterer phase. The radiation loss rates (*γ*_*cw/ccw*_) and coupling rate to waveguides (*γ*_*c*_) are pre-set by the waveguide geometry (grey). For simplicity, ${\epsilon }^{{Sym}}$ in Eq. (1) is marked as $\epsilon$_1_. $\epsilon$_2_ and ${\epsilon }{\scriptstyle{{\prime}\atop{2}}}$ are correspond to ${\epsilon }_{{ccw}\to {cw}}^{{Asym}}$ and ${\epsilon }_{{cw}\to {ccw}}^{{Asym}}$, respectively. **c** Mode splitting versus perturbation strength offset ($\Delta \epsilon$) between the Mie scatterer pair and inter-scatter phase (${\Delta }_{{\varphi }_{v}}$). **d** Enhancement factor (EF) from the chiral EP maximized with the coupling rate to loss rate ratio. Compared to the resonance tuning in diabolic point (DP), the highly localized heater efficiently tunes the coupling strength and cavity lifetime, and thus the peak transmission. The upper bound of chiral contrast between CW and CCW excitations is set by EF. **e** Experimentally measured chiral response with the same time-varying phase, with EF up to 17 dB. **f** The tunable chiral EP facilitated investigations (red), compared to studies allowed in passive systems (unstable or not tunable)

The enhanced response of chiral EPs to small perturbations makes them appealing for sensing^26,27^, efficient electro-optic signal transduction, optical interconnects, and isolators^28,29^. However, current implementations suffer from mechanical instabilities and fabrication-related imperfections. Overcoming these challenges and building mechanically stable chiral EP systems will allow precise control of critical parameters and enable the exploration of enhanced non-Hermitian photonic systems. To address these opportunities, we have fabricated a silicon photonic micro-ring resonator (MRR) with two lithographically defined asymmetric Mie scatterers (Fig. 1a). The two scatterers are geometrically engineered to introduce the same reflectance to the guided modes in one direction (Fig. 1b). By tuning only one of the two optical paths between the asymmetric scatterers with a highly localized heater, which is carefully aligned to one optical path along the resonator, we can control the intra-resonator coupling coefficient between the CW and CCW modes and thus move the system to an EP or away from it. As a result, we deterministically tune chirality, that is the direction of rotation of the optical field inside the resonator (Fig. 1c). We note that in this process, the lithographically defined scatterers redistribute the input optical power from transmission to reflection ports through coupling to the counter-propagating modes in the resonator, without introducing significant loss (Fig. 1c).

The realized non-Hermitian device thus can be deterministically steered between its EP degeneracy (i.e., the field inside the resonator is chiral and propagates in either the CW or the CCW direction and no mode-splitting in the spectra) and non-degenerate states (i.e., both CW and CCW propagating modes exist in the resonator and transmission spectra show mode-splitting). This dynamic control allows us to observe chiral EP which, together with EP-enhanced response of the system to small perturbations, improves phase-amplitude tuning sensitivity and leads to chiral nonlinear and modulation response (i.e., different responses for inputs in the CW and CCW directions). This is attributed to the fact that the asymmetric coupling between CW and CCW modes results in asymmetric field enhancement factors (EF), defined as the ratio of the inter-mode (CW-to-CCW or CCW-to-CW) coupling strengths to the total loss of the modes (Fig. 1d), which in turn leads to different nonlinear resonance shifts for CW and CCW inputs, and hence nonreciprocal response. The difference in the temporal dynamics and response of the system for CW and CCW inputs is clearly seen in Fig. 1e. In our device, we can achieve asymmetric electro-optic modulation at GHz speeds with 17 dB contrast when the system is at the chiral EP, that is the light input in the CCW direction is modulated more than the light input in the CW direction.

Beyond electro-optic tuning and modulation, we also explored the EP’s contribution to the chiral nonlinear response and nonreciprocities. The power range of such nonreciprocal switches is controlled by the chirality and intrinsic loss rate of the resonator (Fig. 1f), which can surpass the power range-transmission trade-off associated with single nonlinear resonators^30,31^. Low-energy and small-footprint silicon microring modulators are widely adopted for optical interconnects and neuromorphic computing^32–36^. If successful, this low-power and precision nanophotonic engineering can reduce the module redundancy in large-scale photonic integrated circuits for interconnects and computing.

## Results

Non-Hermiticity is introduced into our waveguide-coupled microring resonator (MRR) system through asymmetric coupling between its frequency degenerate CW and CCW modes which is controlled by tuning the optical path length between two asymmetric Mie scatterers (i.e., inter-scatterer phase) via thermo-optic effect (Methods). The scatterers are lithographically defined within the resonator mode volume with dimensions between 1/6 and 1/3 times the effective wavelength in the single-mode silicon photonic waveguide. The geometric asymmetry of the scatterers is essential to realize chiral EP in a single MRR. Previously^37^, we compensated asymmetric reflections of distributed Rayleigh scatterers (i.e., in the form of surface roughness or structural inhomogeneity formed during nanofabrication) by introducing a symmetric scatterer in the MRR and demonstrated back-reflection suppression and the emergence of an EP. Here we carefully designed the asymmetric scatterers to provide sufficient contrast between the reflection coefficients for CW and CCW propagating modes, while keeping the quality factors around 10^4^ (inset of Fig. 1a). The scatterer-induced coupling strength of the field into the same or the counterpropagating mode are described as different complex-valued elements (*ϵ*_*2*_ and *ϵ*_*2*_’, marked in the inset of Fig. 1a). Based on two-mode approximation and coupled mode theory, the effects of the two Mie scatterers and the inter-scatterer phase (${\varDelta \varphi }_{v}$) on the total Hamiltonian of the non-Hermitian MRR can be described as^38,39^:

1 $${\rm{H}}=\left(\begin{array}{cc}{{{\Omega }}}_{0}+\varDelta {{{\Omega }}}_{\epsilon {{\_}}{cw}}+\varDelta {\omega }_{v} & {\epsilon }^{{Sym}}+{\epsilon }_{{ccw}\to {cw}}^{{Asym}}{e}^{-j({\varphi }_{0}{+\varDelta \varphi }_{v})}\\ {\epsilon }^{{Sym}}+{\epsilon }_{{cw}\to {ccw}}^{{Asym}}{e}^{j({\varphi }_{0}{+\varDelta \varphi }_{v})} & {{{\Omega }}}_{0}+\varDelta {{{\Omega }}}_{\epsilon {{\_}}{ccw}}+\varDelta {\omega }_{v}\end{array}\right)=\left(\begin{array}{cc}{\chi }_{11}+\varDelta {\omega }_{v} & {\chi }_{12}^{v}\\ {\chi }_{21}^{v} & {\chi }_{22}+\varDelta {\omega }_{v}\end{array}\right)$$

Here ${{\Omega }_{0}={\rm{\omega }}}_{0}-{\rm{i}}{\gamma }_{t}$ is the complex resonance frequency of the unperturbed (without the scatterers) MRR where ${\gamma }_{t}$ denotes the total loss, including the intrinsic (i.e., material, scattering, and radiation losses) and waveguide-resonator coupling losses, ${\varDelta \Omega }_{{\epsilon }_{{cw}/{ccw}}}={\varDelta \omega }_{{\epsilon }_{{cw}/{ccw}}}-i{\gamma }_{{\epsilon }_{{cw}/{ccw}}}$ denote complex frequency change induced by the Mie scatterers in CW/CCW modes, and ${\Delta }_{{\omega }_{{EO}}}={\Delta }_{{\varphi }_{v}}c/L$ is the frequency shift due to the local phase shift ${\varDelta \varphi }_{{EO}}$ where *c* is the velocity of light in the waveguide and *L* is the perimeter length of the MRR. ${\epsilon }^{{Sym}}$ and ${\epsilon }^{{Asym}}$ in the off-diagonal elements of the Hamiltonian represent the complex coupling coefficients induced by the Mie-scatterers between CW and CCW modes and they are set by the geometry of the scatterers (Inset of Fig. 1a). The inter-mode coupling rates defined as ${{\chi }_{12}^{v}=\epsilon }^{{Sym}}+{\epsilon }_{{ccw}\to {cw}}^{{Asym}}{e}^{-j({\varphi }_{0}{+\varDelta \varphi }_{v})}$ and ${{\chi }_{21}^{v}=\epsilon }^{{Sym}}+{\epsilon }_{{cw}\to {ccw}}^{{Asym}}{e}^{j({\varphi }_{0}{+\varDelta \varphi }_{v})}$ can be tuned by varying the inter-scatterer phase ${\varphi }_{0}{+\varDelta \varphi }_{v}$ (Fig. 1b). Note that in Fig. 1, we have marked ${\epsilon }^{{Sym}}$, ${\epsilon }_{{ccw}\to {cw}}^{{Asym}}$, and ${\epsilon }_{{cw}\to {ccw}}^{{Asym}}$ as $\epsilon$_1,_ $\epsilon$_2_ and ${\epsilon }_{2}^{{\prime} }$, respectively. The eigenvalues of $\text{H}$ in the absence of any external thermal effect are ${\omega }_{\pm }={\omega }_{0}+\varDelta {\omega }_{\epsilon }-i\varGamma \pm \xi /2$ where $\xi =\sqrt{4{\chi }_{12}^{v}{\chi }_{21}^{v}}$ is the amount of mode-splitting. This expression assumes that the scatterer-induced frequency shifts are independent of input excitation, thereby preserving transmission reciprocity in the linear regime. Moreover, it implies that by precisely tuning the local phase difference ${\varDelta \varphi }_{v}$, we can steer the system to or from an EP ($\xi =0$) (Supplementary Section 1). Other losses, such as ring-waveguide coupling losses (${\gamma }_{c1/c2}$ in Fig. 1a) and radiation losses (${\gamma }_{{cw}/{ccw}}$ in Fig. 1a) and thus ${\gamma }_{t}$, remain unchanged during the phase tuning.

Phase-only tuning affects the diagonal elements of *H* in the same way as the term ${\varDelta \omega }_{{EO}}$ whereas its effect on the off-diagonal elements differs significantly through the terms ${e}^{\pm j({\varphi }_{0}{+\varDelta \varphi }_{v})}$. Moreover, it drifts the system away from the critical coupling condition and enhances the amplitude tuning efficiency (named optical modulation amplitude, or OMA)^40,41^. The enhanced OMA is evidenced by the large transmittance contrast between the initial and final states at the optimized detuning. The dependence of the resonance frequency shift and the amount of EP-enhanced mode-splitting on ${\Delta \varphi }_{v}$ suggests magnifying the phase-peak amplitude tuning by varying $\Delta \varphi$ (left red part in Fig. 1d). In this way, the amplitude modulation efficiency of the non-Hermitian MRR can be made to exceed that of regular MRR (no peak tuning) and of an MRR operating near the diabolic point (DP). In addition, the chiral response allows opposite tuning effects for light input in opposite directions (right grey part Fig. 1d): At a selected laser-cavity detuning, the electro-optic phase tuning results in enhanced amplitude response in one direction and minimized OMA in the other direction. Thus, the device functions as a chiral electro-optic modulator.

**Fig. 2:**
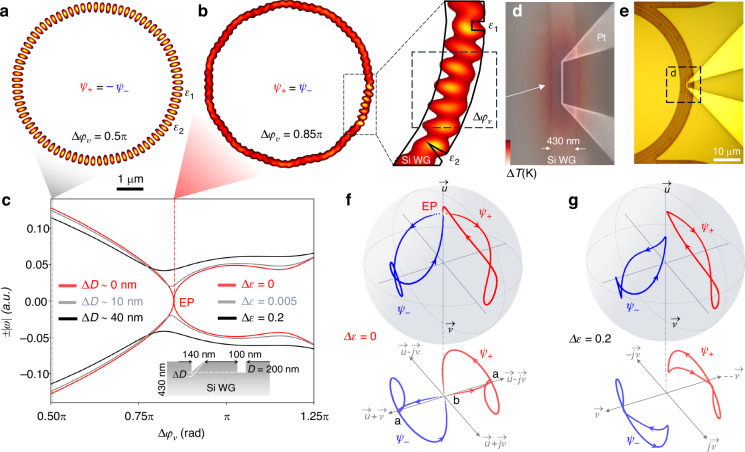
Achieving EP with matching perturbation strength between a pair of Mie scatterers and deterministic tuning across EP with a nanoscale local heater. Asymmetric Mie scatterers (inset of Fig. 1d) pinned mode profiles of a micro-ring, at **a**. a non-EP state (${\varDelta }_{{\varphi }_{v}}=0.5\pi$, *Γ* ~ 1, *S* ~ 0.06) and **b** an EP state ((${\varDelta }_{{\varphi }_{v}}=0.85\pi$, *Γ* ~ 0.064, *S* ~ 1). Right inset: a closer view of the mode profile super-imposed on the Mie scatterers that supports EP. **c** Mode splitting versus inter-scatter phase, with the Mie-scatterers geometry achieving EP (red, $\varDelta \epsilon$ = 0), Mie-scatterer perturbation offset of $\varDelta \epsilon$ = 0.005 (correspondent to the notch depth offset *∆D* ~ 10 nm) and $\varDelta \epsilon$ = 0.2 (*∆D* ~ 40 nm). Inset: illustration of geometric offset ∆*D* given the scatterer and waveguide widths, for the 250 nm SOI. **d** Scanning electronic microscope image of the local heater superimposed with the resulting temperature distribution, illustrating the localized thermo-optic phase shift. **e** Optical microscope image of a microring with a local heater. **f** Representation of the evolution of the eigenvectors on the Bloch sphere by tuning inter-scatterers phase *φ*_*v*_ only. The bottom insects are their projected trajectories for improving clarity. The Mie scatterers combination supports EP ($\varDelta \epsilon$ = 0), and **g** does not support EP ($\varDelta \epsilon$ = 0.2). $\vec{u}$*:* eigenvector for the CW mode, and $\vec{v}$*:* CCW mode. f and g have the same coordinates, and the in-plane ones for (**g**) is simplified for clarity

The design concept is numerically illustrated in Fig. 2a–c. The system can be switched from the non-EP state (mode-splitting with standing wave mode profile in Fig. 2a) to the EP state (traveling wave mode profile in Fig. 2b) when ${\Delta \varphi }_{v}$ is changed from $0.5{\rm{\pi }}$ to $0.85{\rm{\pi }}$ (marked in Fig. 2c). If the geometry of the scatterers (${\epsilon }^{{Sym}}$ or ${\epsilon }^{{Asym}}$) is optimally selected using the optical impedance matching method, then one can continuously vary the inter-scatterer phase ${\Delta \varphi }_{v}$ to drive the system towards an EP (detailed inter-scatterer mode profile in the right inset of Fig. 2b), where both eigenmodes coalesce and feature a square root dependence on detuning ($\left|\Delta \omega \right| \sim \left|\sqrt{\epsilon }\right|$). We quantify whether the system is at the EP degeneracy or detuned from it using the standing wave ratio *Γ* and the mode non-orthogonality parameter *S* (definitions are given in equations S.2-1 and S.2-2, respectively). Near the EP degeneracy, we find *Γ*~0.064 and *S* ~ 1 (derived from Fig. 2b) which imply that the two eigenvectors become collinear and the field inside the resonator is dominantly in one direction (i.e., standing wave ratio close to zero implies a traveling field) as the system approaches the EP. Experimentally, the inter-scatterer phase tuning is achieved by a local heater defined along the perimeter of the MRR. With calibrated electronic characteristics of the sub-µm width heater (Figs. 2d and S2a), the generated temperature profile for optical phase shift is confined within a few µm range between the scatterers defined on the perimeter of the MRR (Fig. 2e). Varying the inter-scatterer phase ${\Delta \varphi }_{v}$ tunes the eigenvalues (Fig. 2c) and the associated eigenvectors (${\psi }_{\pm }$) (Fig. 2f, g). The eigenvectors collapse in the CW direction at the phase matching point (reference point of $\varDelta \epsilon$ = 0). For a scatterer geometry with $\varDelta \epsilon$ ≠ 0, neither the eigenvalues nor eigenvectors cross each other when the inter-scatterer phase is varied (black curve in Fig. 2c and g).

**Fig. 3:**
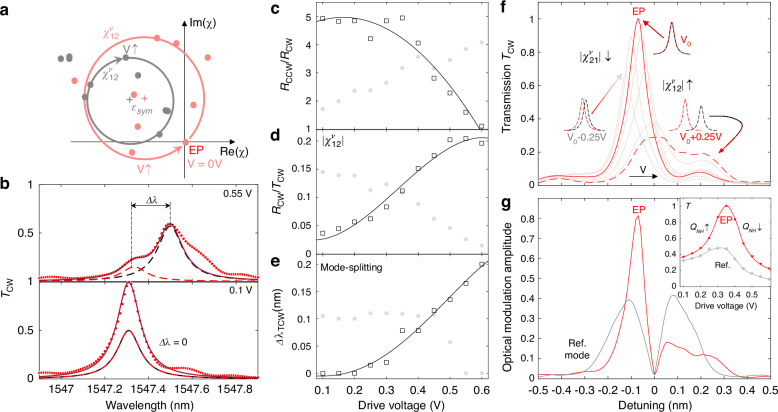
Deterministic tuning of inter-scatterer local phase across EP. **a** Evolution of the off-diagonal elements (${\chi }_{21}^{v}$ and ${\chi }_{21}^{v}$) as a function of the inter-scatter phase. The centers of the circles represent the complex coupling coefficient ${{\rm{\epsilon }}}^{{Sym}}$. The center’s offset is attributed to the surface roughness. The radius is $|{{\rm{\epsilon }}}_{{\rm{ccw}}\to {\rm{cw}}}^{{Asym}}|$ for ${\chi }_{21}^{v}$ circle, and $|{{\rm{\epsilon }}}_{{\rm{ccw}}\to {\rm{cw}}}^{{Asym}}|$ for ${\chi }_{21}^{v}$ circle. EP is achieved at |${\chi }_{12}^{v}$| = 0, as ${\chi }_{21}^{v}$ never crosses the origin. **b** Transmission spectra of a mode at two different voltages applied to the heater to tune the inter-scatter phase. The red dots are experimental data, which are analyzed by the coupled mode theory and empirical model of double Lorentzian fitting for deconvoluting the two dressed states. The additional side peak (near 1547.6 nm) might be from the reflections from bus waveguide terminals. **c** Reflection intensity contrast for CW and CCW excitations. **d** Normalized reflection strength to transmission. **e** Mode-splitting observed in the transmission spectra (in (**a**) and (**b**)) as a function of the drive voltage. Empty squares: experimental data of the target mode in (**a**). Grey solid dots: an alternative mode of the same MRR for comparison. Curves are eye guides. **f** Electro-optic tuning enhancement through coordinated diagonal and off-diagonal tuning. **g** Contrast of transmission |T(0.35 V)/T(0 V)| of a chiral mode (red) and a reference mode (grey) for the same resonator. Inset: Transmission versus drive voltage for the chiral mode (red) and the reference mode (grey)

We performed experiments and evaluated the performance of the phase-only tuning scheme for non-Hermiticity. The results of the experiments are given in Fig. 3. The center resonance wavelength of both CW and CCW excited modes linearly shifts with ${\varDelta \varphi }_{v}$, but the off-diagonal elements move in opposite directions on the complex plane (Fig. 3a). By simultaneously fitting the coupled mode theory (CMT) to the experimentally measured transmission and reflection spectra (Supplementary Section 3), we extracted the voltage-dependent (i.e., voltage applied to the heater) ${\chi }_{12}^{v}$ and ${\chi }_{21}^{v}$ red and grey circles in Fig. 3a). Initially, the system is at an EP, as ${\chi }_{12}^{v}$ locates at the origin and ${\chi }_{21}^{v}$ ≠ 0. As the voltage increases, ${\chi }_{12}^{v}$ rotates in the CCW direction, but ${\chi }_{21}^{v}$ rotates in the CW direction. On the transmission spectra, the mode-splitting increases with the amplitudes of the off-diagonal elements, and the non-zero phase of those off-diagonal elements lead to asymmetry in the deconvoluted modes (red and black dashed curves in Fig. 3b). For an exemplary mode centered near 1547 nm, we observe that the system, which is at the EP when the voltage applied to the heater is 0.1 V (1.5 mW of heating power), moves away from the EP as the applied voltage is gradually increased to 0.55 V (local heating power around 4.5 mW), resulting in an asymmetric mode-splitting. The dynamic tunability around EP (at low drive voltages of less than 0.2 V) is evident by the high reflection contrast, weak reflection with CW excitation, and zero mode-splitting in the transmission spectra obtained for CW excitation (dark squares in Fig. 3c–e). The mode splitting (*Δλ*) is obtained by fitting the measured transmission spectra with a dual Lorentzian model. The full width at half maximum (FWHM) of the decomposed Lorentzian spectra for CW and CCW modes (red and black dashed curves in Fig. 3b) remains unchanged when the voltage applied to the heater is varied, indicating that the process does not change the loss rates (*γ*_*CW/CCW*_) of CW and CCW modes. As a result, the quality factor remains ~10^4^. In the same device under the same electrical drive, the other mode centered at 1542 nm (mode 2) evolves to the EP degeneracy when the voltage is increased from 0.25 V to 0.55 V (Fig. S2b-c, and grey dots in Fig. 3c–e).

We quantify the peak transmission contrast using (see derivation in Supplementary Section 4)

2 $$\frac{{T}_{{EP}}}{{T}_{{non}-{EP}}}={\left|1+\frac{{\chi }_{12}^{v}{\chi }_{21}^{v}}{{\gamma }_{t}^{2}/4}\right|}^{2}$$

which suggests that the contrast can be maximized by precisely tuning the inter-mode coupling strength via ${\varDelta \varphi }_{v}$ if the total loss does (*γ*_*t*_) not vary significantly during the process. We observe that during the thermal tuning of ${\varDelta \varphi }_{v}$, the waveguide-coupled resonator system moves from the under-coupling regime towards critical coupling at the EP degeneracy (*Δλ* = *0)*_,_ resulting in the highest transmission peak (red solid curve in Fig. 3f, at 0.35 V). As the system deviates from the EP, we observe a significant reduction in peak transmission and an increase in the linewidth. We also observed that OMA near the EP (red curve in Fig. 3g) is twice as a non-Hermitian mode (inset of Fig. 3g), under the same initial and final drive voltages.

**Fig. 4:**
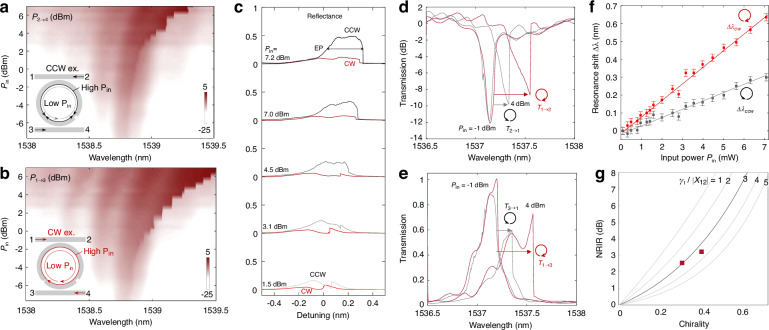
Nonlinearity manifested chirality and nonreciprocity. **a** Input power-dependent transmitted output spectra, with CCW excitation, and **b** CW excitation, at increasing input optical power (*P*_*in*_). Insets: mode evolution at low (dashed) and high excitation powers (solid), for CCW (in (**a**)) and CW (in (**b**)) excitations. **c** Correspondent reflection spectra for CW (red) and CCW (grey) excitations, show expanded EP bandwidth through optical bistability. **d** Nonreciprocal transmission between through ports (1 and 2). **e** Transmission between drop ports 1 and 3 with CW (red) and CCW (grey) excitations. Their transmission spectra nearly overlap at low power (*P*_*in*_ = -1dBm) but have divergent optical nonlinear responses (*P*_*in*_ = 4 dBm). **f** Nonlinear optical resonance shifts versus input optical power with CW (red) and CCW (grey) excitations. **g** The dependence of NRIR on the MRR chirality

The presented low-loss chiral micro-ring resonator facilitates nonreciprocal signal routing in the nonlinear regime. The asymmetry in the field strengths for CW and CCW mode is manifested through asymmetric nonlinear cavity built-up (Fig. 4a–c). Figure 4a–b shows the measured spectra of output power for increasing input power for CCW and CW excitations. The shift in the resonance wavelength for CW excitation (${\varDelta \lambda }_{{cw}}$) for increasing input power exceeds the one for CCW excitation (${\varDelta \lambda }_{{ccw}}$). A similar contrast of the nonlinear resonance shift is observed at the reflection ports (Fig. 4c) where we also see that optical bistability broadens the EP spectral range. To verify the nonreciprocal response, we compare the nonlinear transmission spectra *T*_*1-2*_ (measure port 2 for input at port 1 for CW excitation), and *T*_*2-1*_ (measure port 1 for input at port 2 for CCW excitation) (Fig. 4d). The nonlinearity-induced resonance shifts (Δ*λ*) is proportional to the average intracavity field intensity or cavity energy^42,43^ (Fig. 4d), so is the non-reciprocity intensity range (NRIR) given by:

3 $${NRIR}=\frac{{\gamma }_{t}^{2}/4+{\left|{\chi }_{21}^{v}\right|}^{2}}{{\gamma }_{t}^{2}/4+{\left|{\chi }_{12}^{v}\right|}^{2}}$$

The nonlinear resonance shifts of the CW mode (${\varDelta \lambda }_{{cw}}$) exceeds the one for CCW excitation (${\varDelta \lambda }_{{ccw}}$) (Fig. 4f). Similar contrast of the nonlinear resonance shift is observed between ports 1 and 3 (Fig. 4e). Near EP (${\chi }_{12}^{v}\cong 0$), the loss-limited maximum NRIR is found as $1+\frac{{|{\chi }_{21}^{v}|}^{2}}{{\gamma }_{t}^{2}/4}$. The nonlinear chiral MRR is not subject to the loss-NRIR trade-off which limits the performance of typical single nonlinear Fano resonator systems (Fig. S3). Reducing the loss of the asymmetric Mie scatterer and the phase tuner is critical for facilitating a reasonable NRIR for nonreciprocal device performance. Figure 4g presents the theoretical predictions of NRIR versus chirality for various values of ${\gamma }_{t}/|{\chi }_{21}^{v}|$ together with the experimentally measured NRIR (tracked by comparing the reflection spectra) values. The NRIR can be further improved through finer tuning of the inter-scatter phase or reducing fabrication imperfections.

**Fig. 5:**
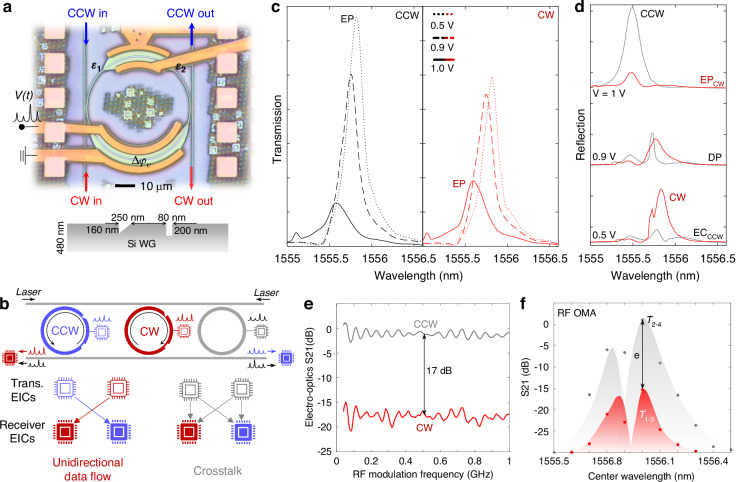
EP enhanced GHz chiral electro-optic modulator. **a** Top view of the non-Hermitian photonic modulators coupled to add/drop bus waveguides embedded in oxide, manufactured through a foundry wafer run. Inset: scatterer designs for the 220 nm SOI. **b** Exemplary application scenario of the chiral electro-optic response enabled unidirectional data flow between transmitting electronic integrated circuits (EICs) and receiving EICs. In conventional MRMs, duplicated circuit topologies for transmitting and receiving terminals are required to avoid crosstalk (grey). **c** Measured transmission spectra at CCW excitation (left) and CW (right) excitation with increasing electrical bias voltage across the *p-n* junctions of GHz phase modulators. The bias-dependent free-carrier absorption modulation is superimposed on the non-Hermiticity induced amplitude tuning response (illustrated in (**b**)). **d** Correspondent reflection spectra, implying the chirality of the device. **e** Electro-optic S21 (normalized OMA measured at high speed) versus electrical modulation speed for CW (red) and CCW (grey) excitations. **f** S21 versus wavelength for CW and CCW excitations

We have also demonstrated wafer-scale manufacturing of doped silicon photonic MRMs (Fig. 5a). The design concept, in particular the asymmetric Mie-scatterer and local phase shift, was first implemented with e-beam lithography (Supplementary Section 5), and then the chip was sent to standard semiconductor foundries to fabricate *p-n* junctions along the ridge waveguide between the scatterers for ultrafast local phase modulation (up to GHz). This combined with proper photonic engineering enabled us to observe chiral modulation controlled with an electronic drive, that is the optical carrier is modulated only in one direction (CW or CCW excitation) while the transmission from the other excitation direction remains unperturbed.

The directional MRM suppresses the side flow of data in an optical link, reduces the circuit design complexity and duplications, and supports recurrent routing topologies for interconnects and computing (Fig. 5b)^44–46^. When the excitation is in the CCW direction, the EP degeneracy in our system emerges at a lower voltage (0.5 V) and the system moves away from the EP at a higher voltage (1 V) resulting in mode-splitting in the transmission spectra. The bias-dependent chirality is the opposite for CW excitation (Fig. 5c). Time domain modulation confirms the unidirectional electro-optic modulator (Fig. 5d). The small transmission contrast (*T*_*CW*_/*T*_*CCW*_) measured under the steady state condition (Fig. 5c) is magnified under dynamic tuning (*∆T*_*CW*_/*∆T*_*CCW*_). Under the same RF electronic drive and DC bias, a vector network analyzer measured 17 dB contrast at modulation speeds up to GHz (Fig. 5e, f). The asymmetric high-speed OMA (optoelectronic S21) is verified at different detuning between laser and resonance wavelengths, showing the characteristic spectra of an OMA (Fig. 5f). The mode-splitting analysis of transmission spectra is detailed in Supplementary Section 6.

## Discussion

Here we have demonstrated a chiral electro-optic modulator and nonreciprocal router in a silicon photonic platform by embedding low-loss asymmetric Mie scatterers in a microring resonator and by tuning the inter-scatterer optical path (i.e., phase) using a highly localized heater integrated to the resonator. Fine-tuning of the inter-scatterer phase steers the resonator controllably to and away from the EP and tunes the effective photon lifetime (i.e., the inverse of the total loss rate) of CW and CCW modes. We note that intrinsic effective loss rates for CW and CCW modes are given as |*γt* | +$|{\chi }_{12}^{v}|$ and | *γt* | +$|{\chi }_{21}^{v}|$, respectively, and the transmission is maximized at the critical point, where the intrinsic effective loss rate is equivalent to the coupling loss rate.

The EP enhancement on dynamic tunability and nonreciprocity is determined by the Mie scatterer-introduced inter-mode coupling strength versus total loss (from material absorption, radiation, and scattering). The critical dimension of around 50 nm for the tailored scatterers was achieved through a high resolution 193 nm deep UV lithography manufacturing line, with optimized immersion lithography, projection mask optimization, and multi-layer photoresist coating design, followed by fine-tuned dry-etch (developed at AIM photonics). Different from the conventional EP systems, the radiation loss remains invariant with tuning, supported by the directly correlated reflection intensity and transmission mode-splitting. Tuning the optical path difference between the scatterers dynamically tunes the inter-scatterer phase which in turn controls the mode-splitting in different ways for CW and CCW modes. Simultaneous tuning of mode-splitting and resonance collectively enhances the electro-optic modulation response (quantified as OMA). Also, the dynamic tuning of the photon-loss channel expands the efficiency-bandwidth limit of the resonator-based modulator^47,48^. Higher *Q* reduces drive voltage and energy, but the associated photon lifetime sets the upper limit of modulation speed. Dynamic tuning of *Q* typically needs coupled resonators or coupled ring-waveguide schemes^49^. Here we adopted a conventional ridge waveguide phase modulator within a single ring, showing twice as high OMA at the EP compared to a reference mode of the same device that is far from the EP. Our method based on embedding asymmetric Mie scatterers can be extended to other types of modulators (e.g., Mach-Zender modulators) to introduce chirality. The chiral electro-optic tuning observed in our device can be further enhanced by more precise nanophotonic engineering and improved fabrication.

The scalability and applicability of the system are verified through a 300 mm wafer-scale manufacturing, demonstrating GHz electro-optic bandwidth with 17 dB contrast between the modulation amplitudes of CW and CCW excitations. The chiral response of the micro-ring circumvents the fundamental trade-off between insertion loss and the range of powers that support non-reciprocal transmission in Fano resonators, which relies on the feeding port-resonator asymmetric coupling. In our approach, the asymmetric scatter breaks the rotational symmetry of the resonator, and the chirality is manifested through nonlinearity and light-matter interactions for nonreciprocities beyond the loss-dynamic range trade-off in conventional nonlinear resonator isolators^50,51^. The interplay among chirality, non-Hermiticity, and resonance-enhanced nonlinear optical bistability introduces nonreciprocal optical signal routing in the fully passive silicon microring resonator. Such phase-sensitive electro-optic tuning, modulation, and all-optical nonreciprocity are facilitated through the strongly engaged CW and CCW modes. The additional degree of freedom on material absorption might need to be further analyzed by considering a multidimensional Hilbert space^52^.

## Materials and methods

### Nano-heater fabrication

The MRR with local heater is fabricated on a silicon-on-insulator (SOI) from Soitec, having a 250 nm silicon layer and 3 μm buried oxide layer. On top of thick SiO_2_ cladding, we fabricate a micro-heater (5 *μ*m arc-length along the ring perimeter, and 50–920 nm width, with ~5 nm alignment precision to the middle of the 420 nm wide silicon wave under 700 nm oxide cladding). The metal is evaporated (5 nm Ti adhesion layer and 100 nm Pt heater) and the heaters are patterned by electro-beam lithography and double resist lift-off process.

### Foundry-manufactured chiral silicon photonic modulator and measurement

The chiral MRMs were manufactured by AIM photonics through a multi-project wafer run (220 nm SOI). The lateral *p-n* diode configurations were defined by ion implantations: boron for *p*-type and phosphorus for *n*-type. Heavily doped *p* + + and *n* + + regions were used to form Ohmic contact, which connected the doped region through *p*+ and *n+* regions. Vertical *vias* are patterned and etched on cladding oxide for the contact regions, followed by standard aluminum metallization for direct contact with the heavily doped Si regions. The photonic structures were defined by 193 nm deep-ultraviolet photolithography on an 8-inch SOI wafer with a 220 nm device layer, followed by reactive ion etching. Three-step etching leaves silicon wing area for supporting the doping and contacts. A thick oxide cover layer is deposited for metal insulation. The RF-photonic measurements are detailed in a recent work^53^.

## Supplementary information

Supplementary Information

## Data Availability

The data are available upon request.
