# Supplementary material for: Chiral exceptional point enhanced active tuning and nonreciprocity in micro-resonators
Source: Light Sci Appl. 2025 Jan 9;14:45. doi: 10.1038/s41377-024-01686-w (PMC11718208; doi:10.1038/s41377-024-01686-w)
Supplement: Supplementary file 1 — Supplementary Information [file 41377_2024_1686_MOESM1_ESM.pdf]

Supplementary Information for

Chiral exceptional point enhanced active tuning and nonreciprocity in micro-resonators

Hwaseob Lee[1†], Lorry Chang[1†], Ali Kecebas, Dun Mao, Yahui Xiao, Tiantian Li,

Andrea Alù[*], Sahin K. Özdemir[*], Tingyi Gu[*]

[†] Authors contributed equally

Email: tingyigu@udel.edu, sko9@psu.edu, aalu@gc.cuny.edu

Supplementary Text

Sec 1: Design of Mie scatterers for unidirectional coupling

Sec 2: Evolution of the mode profiles across an exceptional point

Sec 3: Inter-scatterer phase-controlled mode splitting

Sec 4: Chiral EP enhanced active tuning

Sec 5: Nanofabrication and characterizations

Sec 6: Nanomanufacturing chiral micro-resonator

Figures S1 to S12

Table S1

Supplementary Section 1: Design of Mie scatterers for low loss & unidirectional coupling

The dimensions of Mie scatterers were selected to provide sufficient coupling strength, without reducing the radiation-limited quality factor of the resonator. Performing three-dimensional full-field simulation and optical impedance matching (detailed in Section S1 of the reference [S1]), we located the embedded Mie scatterers such that exceptional points (EPs) emerge. Full field simulation of microring resonator (MRR) with embedded scatterers design confirms that the transmission spectra obtained for clockwise (CW) and counterclockwise (CCW) excitations are different. The conformal mesh with a spatial resolution (in $\hat{x}$ and $\hat{y}$ direction) less than 1/10 of the Mie scatterer dimension is applied. For the vertical direction ($\hat{z}$), the spatial resolution of the mesh is fixed as 1/5 of the simulated silicon waveguide thickness. The simulated waveguide structure is extended completely through the perfectly matched layer for stable and accurate results. Here we have numerically implemented two types of Mie scatterer combinations achieving EP (Fig. S1a-b). The exemplary symmetric and asymmetric Mie scatterers are illustrated in Fig. S1c-d.

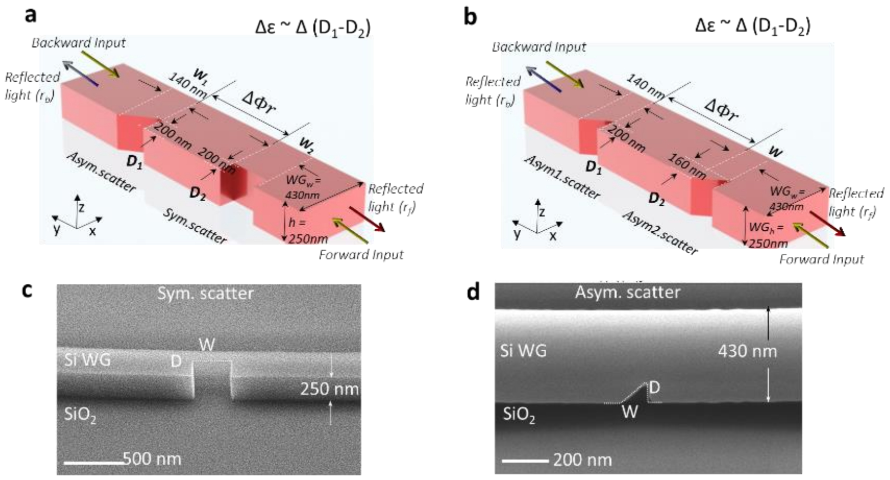

Fig. S1 | Ideal design of the Mie scatterer pair supporting EP (without surface roughness). a. Symmetric and asymmetric. **b.** Two asymmetric ones. The offset between two Mie scatterers is proportional to Δε in Fig. 1. **c.** SEM image of exemplary symmetric scatterer and **d.** asymmetric scatters.

The coupling strength of the i-th Mie scatter-induced scattering into the same ($k = m$) or the counterpropagating ($k \neq m$) mode is described as complex-valued elements ϵ_{ikm} (Fig. S1). The Hamiltonian describing the dynamics of a MRR with two embedded Mie Scatterers as function of the strength of perturbation ϵ_{ikm} induced by the Mie scatterers and the relative phase $\Delta\varphi$ (determined the optical path difference between the scatterers) between them is given by

$$H_2 = \begin{pmatrix} \epsilon_{111} + \epsilon_{211} & \epsilon_{112} + \epsilon_{212}e^{-j\Delta\varphi\text{v}} \\ \epsilon_{121} + \epsilon_{221}e^{j\Delta\varphi} & \epsilon_{122} + \epsilon_{222} \end{pmatrix} = \begin{pmatrix} \Delta\omega_o + \chi_1 & \chi_{12} \\ \chi_{21} & \Delta\omega_o + \chi_2 \end{pmatrix} \quad \textbf{(S. 1-1)}$$

The diagonal elements ϵ_{i11} and ϵ_{i22} determine the resonance frequency shifts induced by the i-th scatters in the CW and CCW modes. The low loss Mie scatter geometries ensure nearly vanished imaginary parts for ϵ_{i11} and ϵ_{i22}, and thus they do not induce additional losses into the CW and CCW modes. The off-diagonal complex elements ϵ_{i12} and ϵ_{i21} determine the i-th scatter induced coupling between CW and CCW modes. The eigenvalues of H_2 are $\omega_\pm = \omega_0 + (\chi_1 + \chi_2)/2 \pm \xi/2$ where $\xi = \sqrt{(\chi_1 - \chi_2)^2 + 4\chi_{12}^v\chi_{21}^v}$. Clearly, $\xi = 0$ results in coalescing eigenvalues (EPs). Corresponding eigenvectors are also computed as: $\psi_\pm = [(\chi_1 - \chi_2 \mp \xi)/2\,\chi_{21} \quad 1]^T$. When $\xi = 0$, both eigenvectors and corresponding eigenvalues coalesce simultaneously, indicating emergence of an EP. In order to achieve EP condition, first, from equation (1), ξ is explicitly expressed in terms of ϵ_{ikm} and $\Delta\varphi$ as: $\xi^2 = (\chi_1 - \chi_2)^2 + 4[\epsilon_{121}\epsilon_{112} + \epsilon_{212}\epsilon_{221} + (\epsilon_{121}\epsilon_{212} + \epsilon_{112}\epsilon_{221})\cos\Delta\varphi + j(\epsilon_{112}\epsilon_{221} - \epsilon_{121}\epsilon_{212})\sin\Delta\varphi]$. For an EP to emerge at $\xi = 0$, the first step is selecting the geometry of the scatterers such that $\epsilon_{112}\epsilon_{221} = \epsilon_{121}\epsilon_{212} = \epsilon'$. With fixed $\text{Im}(\xi^2) = 0$, the mode splitting varies periodically with the electrically tunable $\Delta\phi$. EPs emerge at $\text{Re}(\xi^2) = 0$, where $\cos\Delta\varphi = -\frac{1}{2}\left(\frac{(\chi_1-\chi_2)^2}{4\epsilon'} + \frac{\epsilon_{112}^2+\epsilon_{212}^2}{\epsilon_{112}\epsilon_{212}}\right)$. Therefore, one can conclude that it is possible to realize an EP through tuning the shapes and dimensions of the scatterers and the relative position between them.

Supplementary Section 2: Evolution of the mode profiles across an exceptional point

With a TE mode injected to the waveguide, the magnetic field is perpendicular to the cavity plane and the field can be expanded in cylindrical harmonics (Fig. S2a). The field data in numerical simulation are acquired by having separate spatial distances (L) of 1.4715μm and 1.5915μm between two scatters, corresponding to the optical phase delay 0.85π and 0.5π, computed from: $\Delta\varphi = \frac{2\pi}{\lambda_{eff}} \times L$. This explains that the field can be varied periodically by either physically changing the spatial distance between scatters through an elaborate nanofabrication process or precisely controlling the phase delay by tuning silicon's refractive index (Fig. 2). The field data in the numerical simulation assesses the circularly distributed magnetic field data in the ring resonator when $R = R_o$, shown in Fig. S2b-d. Fig. S2d illustrates the average field intensity along the circular path, where near the degeneracy ($\Delta\varphi\text{v} = 0.85\pi$), the intensity is twice stronger than the intensity extracted from standing mode ($\Delta\varphi\text{v} = 0.5\pi$).

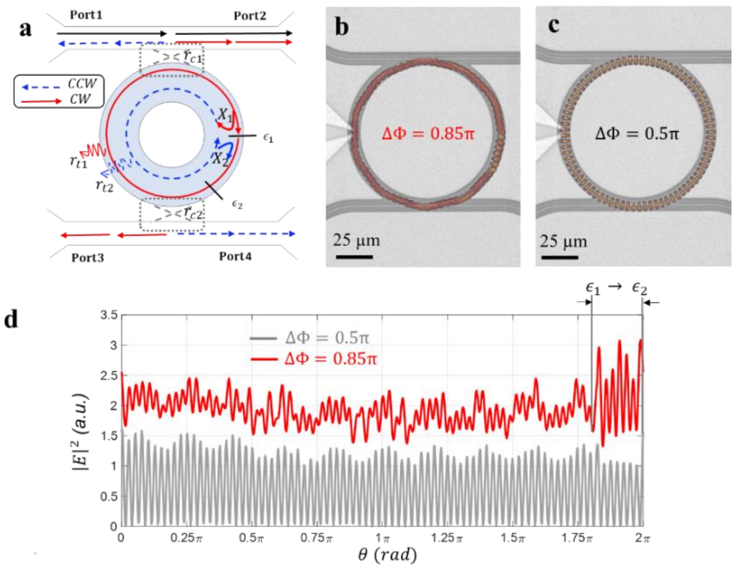

Fig. S2 | Analytical model and numerical simulation. (a) Schematic diagram of two scatterers perturbing the intracavity field of the MRR configured as an add-drop filter. The perturbation strengths of the asymmetric scatters are represented as ϵ_1 and ϵ_2. (b) Traveling mode-like profile with an optimized optical path delay of $\Delta\varphi = 0.85\pi$ provided by a local micro-heater. (c) Standing mode profile with an optical path delay of $\Delta\varphi = 0.5\pi$ provided by a local micro-heater. (d) Mode distribution along the circular direction of ring (θ), for which each field data is extracted alone the center of the waveguide.

Moreover, the mode non-orthogonality and standing wave ratio are computed based on the extracted field data. This computation serves to substantiate the attainability of an exceptional point within our engineered scattering system. The nonorthogonality is quantified by [S2, S3]:

$$S = \frac{|\int dxdy\psi_1^*\psi_2|}{\sqrt{\int dxdy\psi_1^*\psi_1}\sqrt{\int dxdy\psi_2^*\psi_2}} \quad \textbf{(S. 2-1)}$$

where $\Psi_{1(2)}$ is the extracted field data corresponding two eigenmodes of split modes $\omega_{+(-)}$ respectively. S is zero for orthogonal states and it is one for collinear states (e.g., at the EP). The calculated non-orthogonality corresponding to $\Delta\varphi = 0.85\pi$ and $\Delta\varphi = 0.5\pi$ are 1 and 0.401 respectively, which implies that eigenvectors of the system become collinear when $\Delta\varphi$ is properly controlled to steer the system to the vicinity of an EP.

In addition, we find the standing wave ratio [S4]

$$\Gamma = \frac{\sqrt{I_{max}} - \sqrt{I_{min}}}{\sqrt{I_{max}} + \sqrt{I_{min}}} \quad \textbf{(S. 2-2)}$$

as 0.06 and 1 for $\Delta\varphi = 0.85\pi$ and $\Delta\varphi = 0.5\pi$ respectively. In conclusion, this investigation affirms that our meticulously engineered MRR with two Mie scatters can achieve the degeneracy through a judiciously adjusted spatial separation between two scatters or the precise

control of relative phase delay between them. As the system approaches the EP degeneracy, the eigenmodes of the system become non-orthogonal, finally coalescing (becoming collinear) at the EP. Similarly, as the system approaches the EP, the standing wave ratio decreases, becoming zero at the EP (i.e., traveling wave at the EP), This in turn provides a chiral wave propagation in the resonator and leads to maximal field intensity. These numerical results further confirm that the degeneracy obtained by adjusting the optical phase delay between two scatters is indeed an EP [S2, 3].

Supplementary Section 3: Inter-scatterer phase-controlled mode splitting

In general, the micro-heater is introduced in silicon MRR to shift the resonance in different applications such as optical switching and wavelength division multiplexing. Here, a micro-heater is adapted to induce a change in the real part of silicon refractive index through thermo-optic effect and demonstrate the enhancement of resonance shift in Fig. 3 of main text. Here, we discuss the enhancement of resonance shift. The initial (zero bias) Hamiltonian H_0 of a MRR composed of one symmetric and one asymmetric scatterer can be expressed as:

$$H_0 = \begin{pmatrix} \Omega_o + \Delta\omega_\epsilon & \epsilon^{Sym} + \epsilon^{Asym}_{ccw\to cw} e^{-j\Delta\varphi} \\ \epsilon^{Sym} + \epsilon^{Asym}_{cw\to ccw} e^{j\Delta\varphi} & \Omega_o + \Delta\omega_\epsilon \end{pmatrix} \quad \textbf{(S. 3-1)}$$

The two scatterers induce a total resonance shift of $\Delta\omega_\epsilon$ in both CW and CCW modes as well as create asymmetric coupling between the modes. After adding the electro-optic tuning terms, the updated Hamiltonian (H) can be expressed as.

$$H = \begin{pmatrix} \Omega_o + \Delta\omega_\epsilon & \epsilon^{Sym} + \epsilon^{Asym}_{ccw\to cw} e^{-j\Delta\varphi} \\ \epsilon^{Sym} + \epsilon^{Asym}_{cw\to ccw} e^{j\Delta\varphi} & \Omega_o + \Delta\omega_\epsilon \end{pmatrix} + \begin{pmatrix} \Delta\omega_{EO} & \epsilon^{Asym}_{ccw\to cw} e^{-j\Delta\phi_{EO}} \\ \epsilon^{Asym}_{cw\to ccw} e^{j\Delta\phi_{EO}} & \Delta\omega_{EO} \end{pmatrix}$$

$$= \begin{pmatrix} \Omega_o + \chi & \chi_{12} \\ \chi_{21} & \Omega_o + \chi \end{pmatrix} \quad \textbf{(S. 3-2)}$$

where the diagonal elements $\Omega_0 = \omega_o - \frac{j\gamma_t}{2}$ represent the complex energies of the degenerate CW and CCW modes, γ_t representing all losses including both the intrinsic losses (γ_0, including material, bending, ring geometry dependent loss) and the resonator-waveguide coupling losses rates (γ_c). $\Delta\omega_{EO}$ is the resonance shift induced by the external electro-optic tuning. The total resonance shift while a microheater in operation comprises of the absolute value of complex frequency splitting and original resonance shift, which is equivalent to the regular diabolic point MRR ($\Delta\lambda_o = \Delta\lambda_{DP} = \frac{2\pi c}{\Delta\omega_{EO}}$), thus $\Delta\lambda_{tot} = 0.5|\Delta\lambda_{eigen}| + \Delta\lambda_o$, where $\left|\frac{\Delta\lambda_{eigen}}{2}\right|$ is derived from equation (S.3-1):

$$\left|\frac{\Delta\lambda_{eigen}}{2}\right| = \frac{\lambda_0}{\omega_0}|\sqrt{\chi_{21}\chi_{12}}| = \frac{\lambda_o}{\omega_o}\left|\sqrt{\left(\epsilon^{Sym} + \epsilon^{Asym}_{cw\to ccw} e^{j\Delta\varphi}\right)\left(\epsilon^{Sym} + \epsilon^{Asym}_{ccw\to cw} e^{-j\Delta\varphi}\right)}\right| \quad \textbf{(S. 3-3)}$$

Assuming that the intrinsic backscattering perturbation is much greater than the perturbation strength ($\chi_{12} \gg \chi_{21} \sim 0$), the equation shows the resonance shift enhancement is maximized when the system reaches EP (χ_{21}=0).

Supplementary note 4: Chiral EP enhanced active tuning

4.1 Time domain coupled mode theory

Within this section, we will explore the phenomenon of enhanced nonlinearities by leveraging the asymmetry in optical power distribution, supporting the results presented in Fig. 4. Intracavity field intensity-dependent nonlinearity is characterized mainly by the resonance shift, followed by the extinction ratio and line-shape of the transmission spectrum.

We extend the schematics in Fig. S2a with nonlinear effects [S1]. The general coupled-mode theory that governs the two counter-propagating modes (CW and CCW) are:

$$\frac{da_{cw}}{dt} = j(\Delta\omega)a_{cw} - \frac{\gamma_t}{2}a_{cw} - j\chi_{12}^{v}a_{ccw} - \gamma_{c1}\sqrt{P_{CW}} \qquad \textbf{(S. 4-1)}$$

$$\frac{da_{ccw}}{dt} = j(\Delta\omega)a_{ccw} - \frac{\gamma_t}{2}a_{ccw} - j\chi_{21}^{v}a_{cw} - \gamma_{c2}\sqrt{P_{CCW}} \qquad \textbf{(S. 4-2)}$$

where $a_{cw/ccw}$ is the amplitude of the CW and CCW propagating mode; κ is the coupling coefficient between waveguide and cavity, adjusted by the background Fabry-Perot (FP) resonance in the waveguide; $P_{in,cw(ccw)}$ is the incident power; γ_{tot} is the total loss rate that CW mode and CCW mode experience, which are equal when nonlinear effects are ignored; $X_{12(21)}$ is the complex reflection coefficient from the scatters in the resonator, resulting in the modal coupling from CW (CCW) mode to CCW (CW) mode; and $a_{in,\ cw(ccw)}$ are the input optical power injected from Port 1(2) in Fig. S2a. By substituting $a_{cw(ccw)} = A_{cw(ccw)}e^{-j\omega t}$and considering steady state ($\frac{d}{dt}A_{cw(ccw)} = 0$), where $\Delta\omega = \omega_L - \omega_c$ is the detuning between the laser frequency (ω_L) and cold cavity resonance (ω_c).

The transmission spectra of the resonator for CW and CCW excitations can be derived as the following [S1]:

$$T_{cw} = \left|\frac{s_3}{s_1}\right|^2 = \left|\frac{\sqrt{\gamma_{c1}\gamma_{c2}}}{\frac{\chi_{12}^{v}\chi_{21}^{v}}{i\Delta\omega-\gamma_t/2}+i\Delta\omega-\gamma_t/2}\right|^2 \qquad \textbf{(S. 4-3)}$$

$$T_{ccw} = \left|\frac{s_4}{s_2}\right|^2 = \left|\frac{\sqrt{\gamma_{c1}\gamma_{c2}}}{\frac{\chi_{12}^{v}\chi_{21}^{v}}{i\Delta\omega-\gamma_t/2}+i\Delta\omega-\gamma_t/2}\right|^2 \qquad \textbf{(S. 4-4)}$$

As a passive device, the transmission spectra for CW and CCW excitations are nearly the same. Here we explicit the magnified nonreciprocal tuning and nonlinear response as the following.

4.2 Enhanced local phase – amplitude tuning

Near the exceptional point, one of the off-diagonal elements is zero. The transmission

spectrum is written as $T = \left|\frac{\sqrt{\gamma_{c1}\gamma_{c2}}}{i\Delta\omega - \gamma_t/2}\right|^2$, with a peak transmission of $T_0 = \left|\frac{\sqrt{\gamma_{c1}\gamma_{c2}}}{\gamma_t/2}\right|^2$. The local phase tuning deviates the system from EP, reducing the peak transmission of $T_v = \left|\frac{\sqrt{\gamma_{c1}\gamma_{c2}}}{\frac{\chi_{12}^v\chi_{21}^v}{\gamma_t/2} + \gamma_t/2}\right|^2$. The peak transmission contrast (between regular MRM and EP MRM) near EP:

$$\frac{T_0}{T_v} = \left|1 + \frac{\chi_{12}^v\chi_{21}^v}{\gamma_t^2/4}\right|^2 \quad \textbf{(S. 4-5)}$$

Furthermore, from Eq. (S.3-3) it is evident that the relative phase between the scatterers, $\Delta\varphi$, which can be controlled by applying voltage bias, has significant effect on $\Delta\lambda_{eigen}$. This theoretical analysis provides an understanding about the driving mechanism of the enhanced modulation and is consistent with experimental measurements shown in Fig 3.

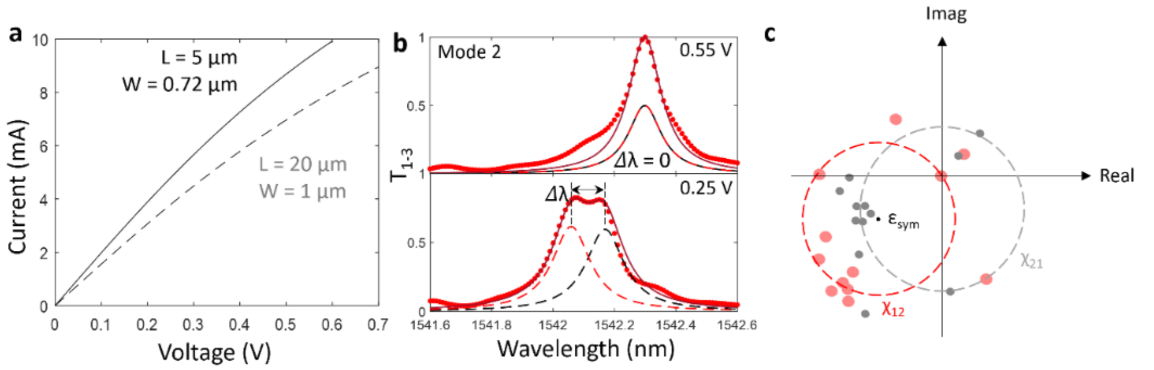

Fig. S3 | Dynamic tuning of Hermiticity with local electro-optic phase shifter. (a) The designed local heater current-voltage characteristics. (b) Transmission spectra of a mode evolve with the driving voltage for the inter-scatter phase. (c) The real part and imaginary part of χ_{12} and χ_{21} for the above transmission spectra.

4.3 Chiral cavity energy built-up

The total photon number in the microring resonator is computed by the sum of the intensities of two eigenmodes $U = |a|^2 = |a_+|^2 + |a_-|^2$ where $a_+ = \frac{a_{cw}+a_{ccw}}{\sqrt{2}}$ and $a_- = \frac{a_{cw}-a_{ccw}}{\sqrt{2}}$. Therefore, the total cavity energy at steady state is given by

$$U_{cw\_in} = \gamma_{c1}A_{cw,in}^2 \frac{\gamma_t^2/4 + |\chi_{12}^v|^2}{\left(\frac{\gamma_t^2}{4} + \chi_{12real}\chi_{21real} - \chi_{12imag}\chi_{21imag}\right)^2 + \left(\chi_{12real}\chi_{21imag} + \chi_{12imag}\chi_{21real}\right)^2} \quad \textbf{(S. 4-6)}$$

when only CW input is present, and by

$$U_{ccw_{in}} = \gamma_{c2} A^2_{ccw,in} \frac{\gamma_t^2/4 + |\chi_{12}^v \chi_{21}^v|^2}{\left(\frac{\gamma_t^2}{4} + \chi_{12real}\chi_{21real} - \chi_{12imag}\chi_{21imag}\right)^2 + \left(\chi_{12real}\chi_{21imag} + \chi_{12imag}\chi_{21real}\right)^2}$$

(S. 4-7)

When only CCW input is present. There is a difference in the energy stored in the resonator for CW and CCW inputs due to the strong coupling and asymmetric coherent interference between CW and CCW modes. As a result, a dissimilar amount of nonlinear resonance shift is observed when transmission spectra for CW and CCW inputs are compared. This in turn leads to asymmetry in the transmission spectra. From (**S.4-3** and **S.4-4**), we can derive the cavity energy contrast of CW and CCW excitations as (γ_{c1}=γ_{c2} in our device):

$$\frac{U_{cw\_in}}{U_{cw\_in}} = \frac{\gamma_t^2/4 + |\chi_{12}^v|^2}{\gamma_t^2/4 + |\chi_{21}^v|^2} \qquad \textbf{(S. 4-8)}$$

Equation (S. 4-7) suggests that the manipulation of power asymmetry in reflection, represented by the system's chirality, allows for the augmentation of nonlinear effects. The nonlinear resonance shift (dominated by the thermal effect in silicon) is proportional to the cavity energy. The ratio between resonance shift and input power has a large contrast for CW and CCW excitations, which originated from the cavity energy difference and chirality of the ring (equation S. 4-5). Such asymmetric field enhancement and cavity energy built-up are experimentally verified through the nonreciprocities and chiral all-optical switching (Fig. 4).

The chiral energy built-up is experimentally characterized in Fig. 4. We characterized the chiral nonlinear response and power-dependent transmission (nonreciprocity between ports 1 and 2 as in Fig. 4d). The non-reciprocity intensity range ($NRIR$ = $\frac{U_{cw\_in}}{U_{cw\_in}}$) remains unchanged with detuning (δ, the ratio between laser-resonance offset and half linewidth of the resonator) (Fig. S4a). NRIR only depends on the asymmetric inter-mode coupling, and increases with chirality (near EP), without additional loss for introducing asymmetric couplings as Fano nonlinear resonator (Fig. S4b). Near EP, the chiral energy built-up/nonlinear transmission shift has a similar form as the EP-enhanced tuning effect: $NRIR = 1 + \frac{|\chi_{12}|^2}{\gamma_t^2/\mathbf{4}}$. An isolation depth of up to 4dB has been achieved so far (Fig. S4c).

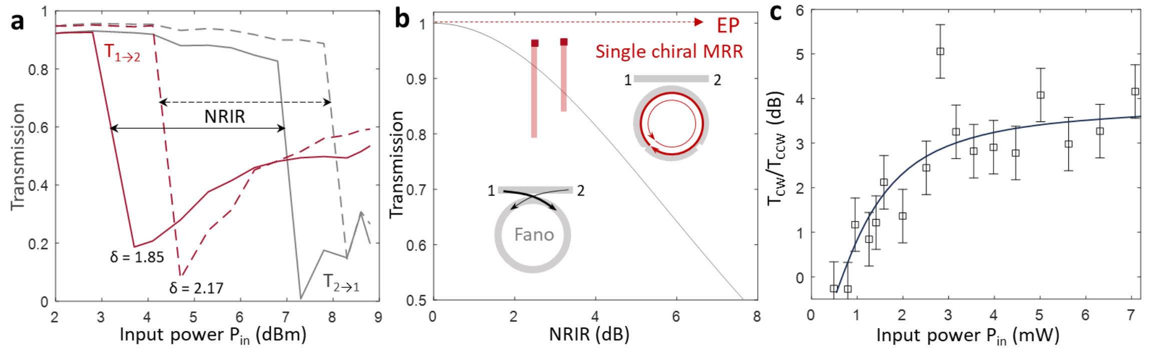

Fig. S4 | High transmission nonreciprocal response near chiral EP. a. Transmission versus input power from port 1 to 2 (red) and from port 2 to 1 (grey) at different detuning. **b.** Transmission versus NRIR for a single chiral (red dashed line) and Fano resonator (grey dashed line). Left inset: asymmetric coupling coefficients between feeding ports and resonator resulted in nonreciprocity in the Fano resonator, which resulted in the trade-off between transmission and NRIR. Right inset: asymmetric coupling between CW and CCW modes resulted in nonreciprocity in a chiral MRR. Lines and curves are theoretical predictions and squares are experimental data. **c.** Transmission contrast between CW and CCW excitations under the same input power. Squares are experimental data, and the curve is an eye guide.

4.4 Comparison between nonlinear CMT and experimental data

The nonlinear time domain CMT (**S.**4-1 ~ 4-2) derives the chiral energy built-up [S5]. The ensuing analysis is performed by Adams-Bashforth-Moulton method [S6].

$$\frac{da_{cw}}{dt} = -(j(\omega_L - \omega_0 + \Delta\omega_{nonlinear}) + \frac{1}{2\tau_t})a_{cw} - j\chi_{12}a_{ccw} - r_{c1}\sqrt{P_1} - r_{c2}\sqrt{P_3} \quad \textbf{(S. 4-9)}$$

$$\frac{da_{ccw}}{dt} = -\left(i(\omega_L - \omega_0 + \Delta\omega_{nonlinear}) + \frac{1}{2\tau_t}\right)a_{cw} - i\chi_{21}^{v}a_{cw} - r_{c1}\sqrt{P_2} - r_{c2}\sqrt{P_4} \quad \textbf{(S. 4-10)}$$

$$\frac{dN}{dt} = \frac{1}{2\hbar\omega_0 n_g{}^2}\frac{\beta_{si}c^2}{V_{FCA}{}^2}(U^2) - \gamma_{fc}N \quad \textbf{(S. 4-11)}$$

$$\frac{d\Delta T}{dt} = \frac{1}{\rho_{si}c_{p,si}V_{cavity}}P_{abs} - \gamma_{th}\Delta T \quad \textbf{(S. 4-12)}$$

where N is the free-carrier density and ΔT is the cavity's temperature shift. The time-dependent resonant shift of the cavity is noted by $\Delta\omega_{nonlinear} = \Delta\omega_N - \Delta\omega_T$, where the free carrier dispersion is $\Delta\omega_N = \omega_0(\xi_e N + \xi_h N^{0.8})/n_{si}$. The thermally induced dispersion is $\Delta\omega_T = \omega_0\Delta T(dn_{si}/dT)/n_{si}$. Kerr dispersion is negligibly small compared to the thermal and free-carrier mechanisms. The total loss rate dependent on input power is written by: $\gamma_t = (\gamma_{c1} + \gamma_{c2} + \gamma_{in}) + (\gamma_{lin} + \gamma_{TPA} + \gamma_{FCA})$, where the linear absorption γ_{lin} for silicon is demonstrated to be small, γ_{in} is the intrinsic loss which is related to the propagation loss, and $\gamma_{c1/2}$ is the coupling loss from the bus and drop waveguide. The free carrier absorption rate is

given by $\gamma_{FCA} = c\sigma N(t)/n_g$. The field-averaged two-photon absorption rate is $\gamma_{TPA} = b_2 c^2/n_g^2/V_{TPA}|U(t)|^2$, where b_2 is the effective two-photon absorption coefficient. The total absorbed power is computed by $P_{abs} = (\gamma_{lin} + \gamma_{TPA}(U) + \gamma_{FCA}(U^2))U$.

TABLE S1 Estimated physical parameters from time-dependent coupled-mode theory-experimental matching, three-dimensional numerical field simulations, and measurement data. τ_{fc} is the effective free-carrier lifetime accounting for both recombination and diffusion.

Parameter	Symbol	Si microring
TPA coefficient	β_2 (10^{-11}m/W)	0.84
Kerr coefficient	n_2 (m^2/W)	0.44×10^{-17}
Thermo-optic coeff.	dn/dT	1.86×10^{-4}
Specific heat	$c_v\rho$ (W/Km^{-3})	1.63×10^{6}
Thermal relaxation time	$\tau_{th,c}$ (μs)	1
Thermal resistance	R_{th} (K/mW)	50
FCA cross section	σ (10^{-22}m^3)	14.5
FCD parameter (electron)	ζ (10^{-28}m^3)	8.8
FCD parameter (hole)	ζ (10^{-28}m^3)	4.6
Carrier lifetime	τ_{fc} (ns)	0.45
Two photon absorption volume	V_{TPA} (10^{-18}m^3)	35.15 [FDTD]
Free carrier absorption volume	V_{FCA} (10^{-18}m^3)	31.7414 [FDTD]
Effective cavity mode volume	V_{cavity} (10^{-18}m^3)	37.7594 [FDTD]

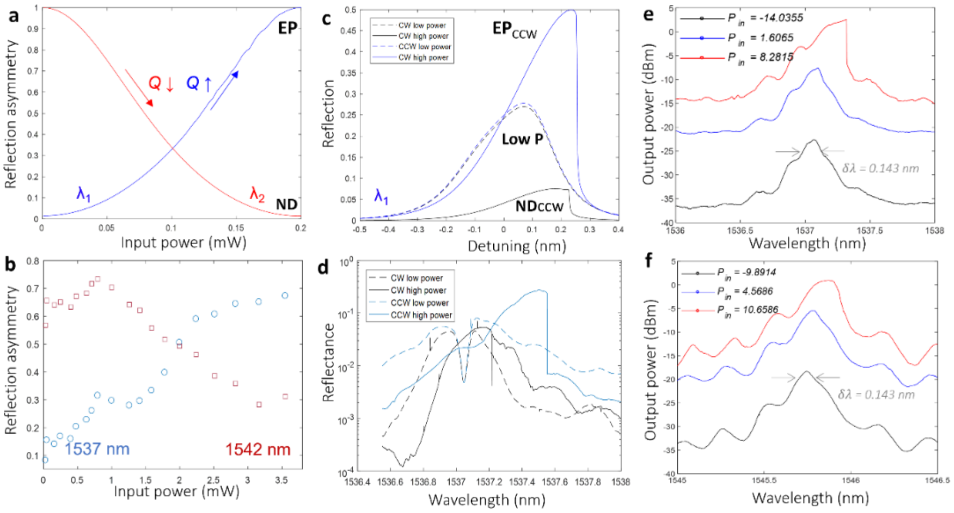

Fig. S5 | Comparison between nonlinear CMT and experiment. (a) Simulated and (b) measured optically tunable reflection asymmetry where reflection asymmetry = $(R_{2-3} - R_{1-4})/(R_{2-3} + R_{1-4})$ for two different resonant modes' cases where the reflection asymmetry

increases with increasing power for λ$_1$ mode (Q_{NH}↑), and it decreases for λ$_2$ mode (Q_{NH}↓). (c) Simulated spectra of one resonance with CW mode evolved from DP to EP and CCW mode evolved from DP and ND with increased input power. Dashed curves: reflection spectra at low power. Solid curves: high power. (d) Experimentally measured spectra for the mode of resonance wavelength around 1537 nm in b. (e) EP and (f) non-EP transmission spectra from low optical power to high optical power with the same quality factor.

Supplementary Section 5: Nanofabrication and Characterizations

5.1 Design and fabrication of the photonic layer

Fig. S6 illustrates the details of the fabrication and characterization of the photonic layer. Fig. S6a shows a dark field image of the chiral microring resonator array with add-drop filters (for obtaining the transmission and reflection in Fig. 3), where an inversely designed Y junction coupler was introduced to measure the CCW light injected reflection spectrums. SEM image of the Y junction shows the well-defined nanostructure in Fig. S6b. The inverse design method is performed based on the ‘adjoint method’ implemented in Python. And measured Y junction performance verifies the ideal 3dB loss as a 50:50 power splitter as shown in Fig. S6d. Those low-loss components are critical for nonlinear measurements, where the transmission is strongly dependent on the input power. The fiber-to-fiber loss of the single-step etched apodised grating coupler (without 3dB beam splitter) is ~10dB (Fig. S6c, d). The grating coupler loss per facet was estimated to be 5 dB near 1539 nm.

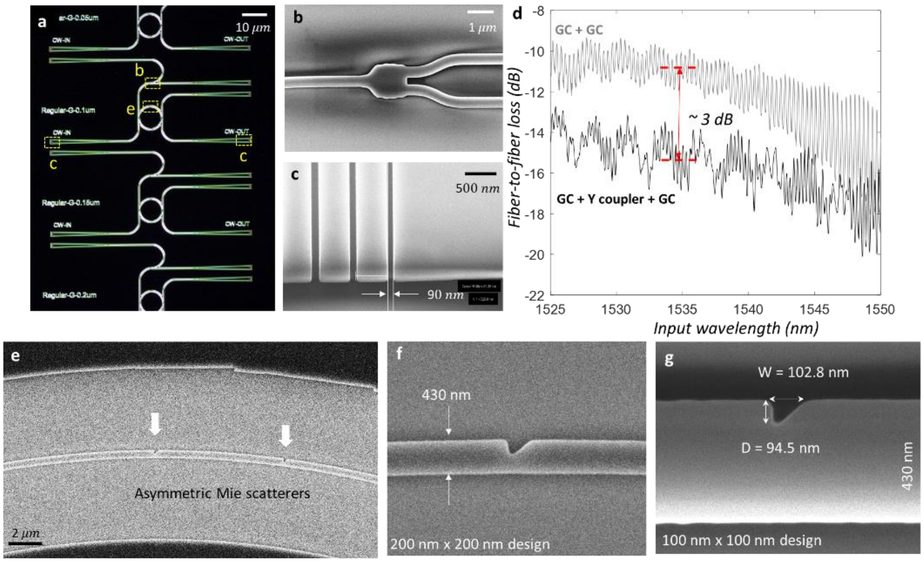

Fig. S6 | Fabricated add-drop filter type ring resonator for obtaining the results in Fig. 3. a. Dark field image of the fabricated photonic layer with add-drop filter around the chiral microring. The geometry of Mie-scatterer pair (embedded in the ring) achieving EP is illustrated in Fig. S1. **b.** SEM image of the inversely designed Y junction coupler, and **c.**

apodised grating coupler, achieving 5dB insertion loss with a single step etch. **d.** Experimentally measured transmission spectrums of grating couplers (GC) with and without Y junction. **e.** The portion of the chiral microring with a pair of Mie scatterers. **f.** Zoom-in image of one design and **g** the other design we used for obtaining the results in Fig. 3.

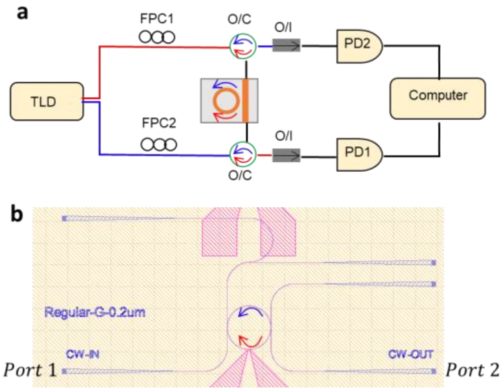

Fig. S7 | Testing setup for the chiral micro-resonator. a. Set up schematics for obtaining the chiral transmission spectra. TLD: tunable laser diode. FPC: fiber polarization controller. PD: photodetector. O/C: switchable optical circulator. The chip-device coupling is unchanged during the measurement. The direction of optical excitation is controlled by the O/C. O/I: optical isolator. **b.** Device layout and testing ports.

Fig. S6e-g illustrates the fabricated asymmetric Mie scatterers embedded in the micro-resonator. We prepared asymmetric Mie scatterers with varying dimensions from 200 nm to 100 nm (Fig. S6f,g). The smaller features around 100 nm show better performance. The design-fabrication variations are characterized to be around 5nm. The offset is affected by the adjacent topologies (Fig. S6g). The chirality measurement setup and nonlinear measurements are carried out using the setup illustrated in Fig. S7.

5.2 Precision alignment of nano-heater to nanophotonic structures

The fabrication flow chart for the nanophotonic layer and subsequent electronic layer is illustrated in Fig. S8a-b. The fabrication on the silicon layer defines the position and geometry of the microring and Mie scatterers (Fig. S8c), and the metal layer is precisely aligned with the photonic layer for achieving localized heating (Fig. S8d-f). Bi-layer resist (CSAR and LOR3A) was used for the better sidewall of the metal electrode than the traditional lift-off process. Electro-beam lithography alignment is a significant step to place the metal on the small area of the waveguide. Under the microscope, we place the Faraday cup to be the center of the optical microscope with the closely adjusted focal length. Then, we normalize the relative position for the Faraday cup and then shift the stage to see the chip in the middle of the microscope. Then we measure the four points of the chip edge in xy plane and calculate the chip position in x-y

direction. These numbers are substituted in EBPG computer before starting the E-beam writing. SEM image of the fabricated microheater in Fig. S8d-f shows the nano heater can be aligned to the waveguide under the 700nm oxide cladding, with an offset of less than 5 nm. The fabricated microheater features a narrowest width of 50 nm with low edge roughness (Fig. S8f). The low edge roughness of the nano-scale metal layer is attributed to the bi-layer resist. We placed the chip in NMP solution for at least 1 hour at 80 degrees for the entire removal of the unexposed areas under E beam writing.

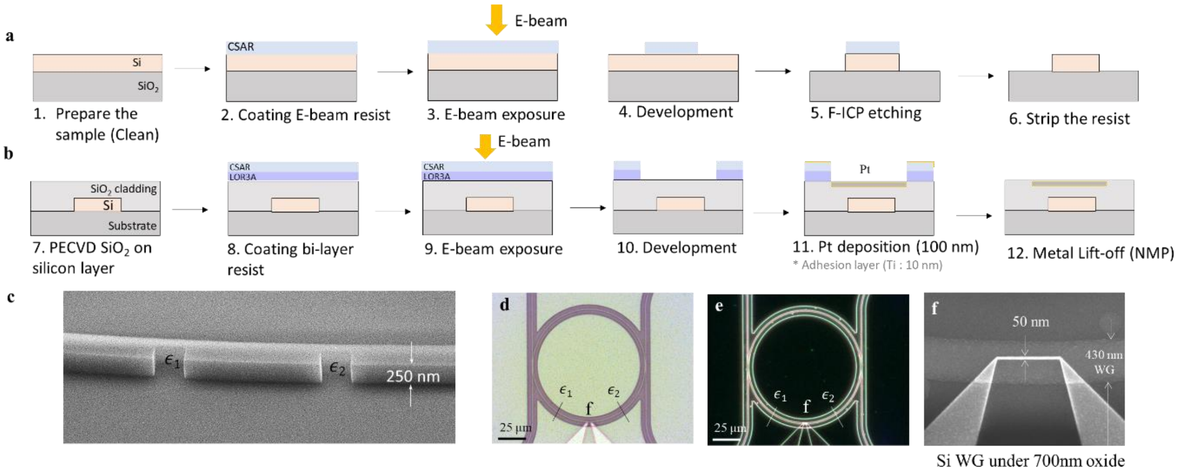

Fig. S8 | Schematic of the active device fabrication process and SEM image of the fabricated narrowest micro-heater on top of the silicon ring resonator. a. Fabrication process of the photonic layer with a single layer resist, and **b**, nano-scale electrodes aligned to the photonic layer, with double layer resist for achieving high precision and nanoscale heater. **c.** SEM image of an exemplary Mie scatterer pair embedded in the microresonator. **d.** Bright-field and **e** dark field image of the chiral microring with integrated heater. **f.** Achieved metal heater was 50 nm, and the heater design-fabrication offset is less than + 15 nm. *The alignment error between the heater and the middle of the waveguide (430nm wide) is less than 5 nm.*

The oxide cladding thickness is optimized by balancing the optical and heater designs. Through numerical full-field optical simulations (Fig. S9a-b), a minimal thickness of 700 nm oxide cladding is selected to prevent the waveguide mode coupling to the metal heater layer. Thinner oxide cladding also facilitates more efficient heating near the silicon waveguide. We characterize the heater designs with different widths. The heating efficiency of $\Delta T/P \sim 9.1$ K/mW is obtained through the current-voltage curve and temperature dependence of the Pt resistivity (Fig. S9c). Note that geometric engineering such as serpentine structures or introducing undercut can improve the heating efficiency, but those structures are too bulky for localized heating. From the thermal simulation (Fig. S9b), the tuning efficiency near the Si waveguide region is nearly half of the one on the top metal layer. The thermos-optic tuning

efficiency is around 4.5 K/mW, which is more than two times the value reported in typical microring heaters (attributed to the nanoscale design) [S7-S8].

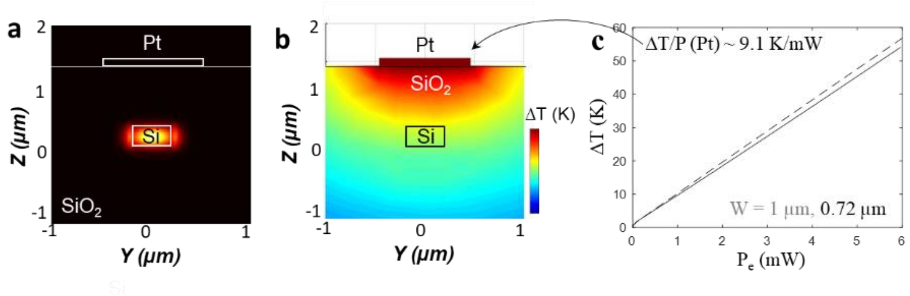

Fig. S9 | Nanoscale local heater design and characterization. a. Simulated optical field confided in the waveguide of the ring and **b,** Simulated temperature distribution from the top Pt heater on SOI substrate. **c.** Measured local temperature versus input power for the nano-heaters. The local temperature is derived from the measured current (I) and voltage (V) curve (Fig. S2a), with temperature dependence of the resistivity. The tuning efficiency on the metal layer is measured to be 9.1K/mW, and the tuning efficiency at the Si waveguide layer is around 4-5 K/mW.

Supplementary Section 6: Nanomanufacturing chiral micro-resonator

6.1 Nanomanufacturing of tunable chiral micro-resonator

With the advancement of technology nodes of the semiconductor photonic foundry, deep UV photolithography can support the nanomanufacturing of the chiral micro-resonator concept, with immersion lithography and fine-tuned etching steps. The critical dimension can reach 50 nm, as shown in Fig. S10a. The intrinsic quality factor of the microring resonator (with minimal surface roughness) decreased from 10^5 to 10^4 with the Mie scatterer (which is likely attributed to mode-splitting rather than additional radiation loss). Tuning such chiral microring towards EP requires additional electronic layers. Both photonic and electronic designs are adjusted and optimized complying with the foundry requirements. Two specially designed Mie scatterers (symmetric + asymmetric in Fig. S10a) with the same depth are utilized here, to ensure the same perturbation strength given the unknown geometric offset. The distance between two scatterers is approximately 30μm hosts the inter-scatterer phase tuner. We implemented structures of micro-heaters (Fig. S10b-c) and local *p-n* junction for non-thermal and localized phase tuning and modulation (Fig. 10d).

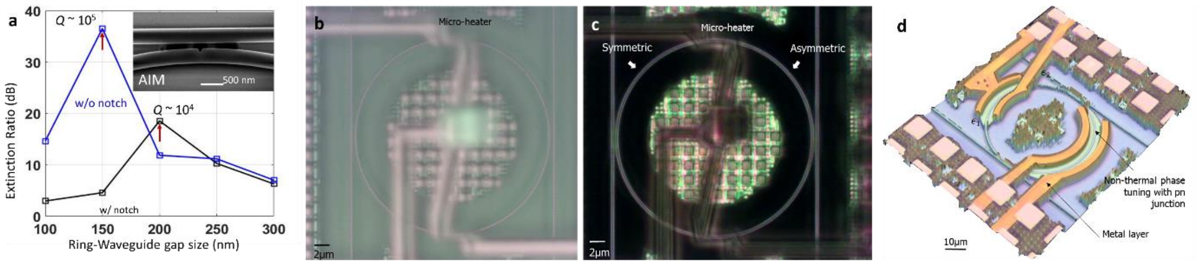

Fig. S10 | Nanomanufactured Mie scatterer loss characterization, with the local phase tuning schemes of the micro-heater and *p-n* junction. (a) The extinction ratio of foundry-manufactured notched MRRs versus ring-WG gap size (black), compared to the same design without notch (blue). Red arrows indicate the critical point. Inset: SEM image of the scatterer fabricated by 193nm deep-UV photolithography. Note that the foundry manufactured MRR has extremely low surface roughness. **b.** Bright field and **c.** dark field image of the chiral microring with a pair of matched Mie scatterers, and local heaters. **d.** Alternative design with inter-scatterer phase tuned by *p-n* junction. The refractive index change through carrier dispersion is highly localized.

For reducing the optical loss, the p-type doping concentration in the chiral microring modulator is 2x10^7 cm^{-3}, while the n-type doping concentration is 6×10^7 cm^{-3}. Heavy and heavily doped regions are utilized for achieving Ohmic contact. Due to the short distance of the *p-n* junction in the chiral microring modulator, the change in the refractive effective index is on the order of 10^{-4}. Additionally, the loss in the waveguide resulting from this structure is approximately 10^{-2} dB. These values feature the sensitivity and efficiency of chiral modulator in terms of its refractive index modulation and the accompanying loss in the waveguide. The estimated total quality factor, determined through a single Lorentzian fit process, is approximately ~10^4. In Fig. S11a, the electro-optical tuning measurements are used to explain the chiral modulation behavior. A specific mode within the brown dashed box from Fig. S11b is selected for analysis. Fig. S11c provides the time domain modulation results at 1555.85nm. With the same electrical drive (black curves), CW (blue) and CCW (red) excitation exhibit distinguished responses, demonstrating directional electro-optic modulation.

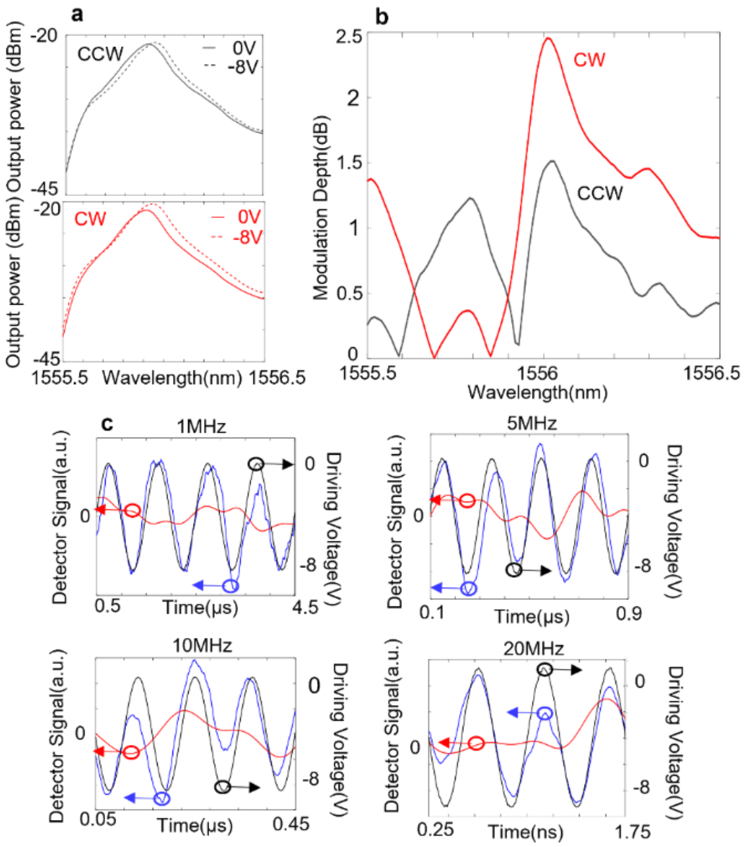

Fig. S11 | Electro-optical tuning analysis and temporal modulation results. (a) DC performance of the CW and CCW reversed biased ring modulator. (b) Modulation depth (OMA) versus wavelength. (c) Time domain experimental results in reversed bias with 1MHz, 5MHz, 10MHz, and 20MHz. Driving signal: black solid line; CW: red solid line; CCW: blue solid line.

6.2 Carrier plasma-induced local phase shift and associated mode splitting

In this study, we utilized double Lorentzian formulas to fit the forward bias experimental data for both CW and CCW excitations, as depicted in Fig. S12. Through the fitting process, we successfully extracted crucial parameters, including the total quality factor (Q_t), intrinsic quality factor (Q_{in}), and coupling quality factor (Q_c) from the obtained results. Additionally, the mechanism of the EP ring resonator is explained using $\frac{1}{Q_t} = \frac{1}{Q_c} + \frac{1}{Q_{in}} + \frac{1}{Q_{NH}}$. For both directions, Q_c and Q_{in} remain unchanged. External bias modifies Q_{in} through carrier absorption and dispersion effect, and Q_{NH} through inter-mode coupling. Q_{in} includes radiation loss and material loss. Further p-n junction engineering can reduce carrier absorption associated Q_{in} reduction, for improved performance with high-speed operation.

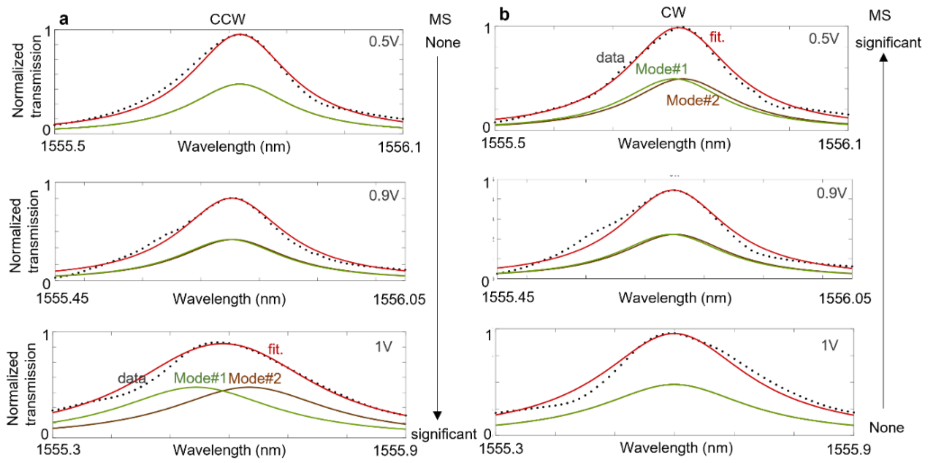

Fig. S12 | Chiral response of mode-splitting on transmission ports. Transmission spectra were obtained for (a) CCW and (b) CW excitations. The dotted line is experimental data. Brown and green solid curves represent the modes inside the resonance. Red solid curves are the convolution of the two Lorentzian components. MS: mode-splitting.

Supplementary information accompanies the manuscript on the Light: Science & Applications website (http://www.nature.com/lsa)

References

[S1] Lee, H. *et al.* Chiral exceptional point and coherent suppression of backscattering in silicon microring with low loss Mie scatterer. *eLight* 3, 20 (2023).

[S2] Wiersig, J., Eberspächer, A., Shim, J. B., Ryu, J. W., Shinohara, S., Hentschel, M., & Schomerus, H. Nonorthogonal pairs of copropagating optical modes in deformed microdisk cavities. *Physical Review A*, *84*, 023845 (2011).

[S3] Wiersig, J., Kim, S. W., & Hentschel, M. Asymmetric scattering and nonorthogonal mode patterns in optical microspirals. *Physical Review A*, *78*, 053809 (2008).

[S4] Saleh, B. E., & Teich, M. C. *Fundamentals of photonics*. john Wiley & sons. (2019).

[S5] Lee, H. Designed non-Hermitian states in notched silicon micorirng resonator, Ph.D. thesis (2021).

[S6] Chiou, J. C., & Wu, S. D. On the generation of higher order numerical integration methods using lower order Adams–Bashforth and Adams–Moulton methods. *Journal of computational and applied mathematics*, *108*, 19-29 (1999).

[S7] Dong, P., Chen, Y.K., Gu, T., Buhl, L.L., Neilson, D.T. and Sinsky, J.H., Reconfigurable 100 Gb/s silicon photonic network-on-chip. *Journal of Optical Communications and Networking*, 7(1), pp.A37-A43 (2015).

[S8] Harris, N. C., Ma, Y., Mower, J., Baehr-Jones, T., Englund, D., Hochberg, M. and Galland, C. Efficient, compact and low loss thermo-optic phase shifter in silicon, *Opt. Express* 22(9), 10487–10493 (2014).
